# Unveiling racial disparities in prostate cancer using an integrative genomic and transcriptomic analysis

**DOI:** 10.1016/j.cellin.2025.100238

**Published:** 2025-02-17

**Authors:** Abdalla Elbialy, Akshay Sood, Shang-Jui Wang, Peng Wang, Ahmed Fadiel, Anil V. Parwani, Steven Huang, Gennady Shvets, Nagireddy Putluri, Jenny Li, Xuefeng Liu

**Affiliations:** a OSU Comprehensive Cancer Center, The Ohio State University, Columbus, OH, 43210, USA; b Department of Urology, College of Medicine, The Ohio State University, Columbus, OH, 43210, USA; c Department of Radiation Oncology, College of Medicine, The Ohio State University, Columbus, OH, 43210, USA; d Department of Medicine, College of Medicine, The Ohio State University, Columbus, OH, 3210, USA; e Computational Oncology Unit, The University of Chicago Comprehensive Cancer Center, 900 E 57th Street, KCBD Bldg., STE 4144, Chicago, IL, 60637, USA; f Departments of Pathology, College of Medicine, The Ohio State University, Columbus, OH, 43210, USA; g School of Applied and Engineering Physics, Cornell University, Ithaca, NY, 14850, USA; h Department of Molecular and Cellular Biology, Baylor College of Medicine, Houston, TX, 77030, USA; i Departments of Pathology, Urology, and Radiation Oncology, College of Medicine, The Ohio StateUniversity, Columbus, OH, 43210, USA

**Keywords:** Racial disparities, Prostate cancer, Genomics, Transcriptomics, Etiology, Differential analysis

## Abstract

Prostate cancer exhibits significant racial disparities, with African American (AA) individuals showing ∼64% higher incidence and 2.3 times greater mortality rates compared to their Caucasian (CA) counterparts. Understanding the complex interplay of genetic, environmental, lifestyle, socioeconomic, and healthcare access factors is crucial for developing effective interventions to reduce this disproportionate burden.

This study aims to uncover the genetic and transcriptomic differences driving these disparities through a comprehensive analysis using RNA sequencing (RNA-seq) and exome sequencing of prostate cancer tissues from both Black and White patients.

Our transcriptomics analysis revealed enhanced activity in pathways linked to immune response and cellular interactions in AA prostate cancer samples, with notable regulation by histone-associated transcription factors (HIST1H1A, HIST1H1D, and HIST1H1B) suggests potential involvement of histone modification mechanisms. Additionally, pseudogenes and long non-coding RNAs (lncRNAs) among the regulated genes indicate non-coding elements' role in these disparities.

Exome sequencing identified unique variants in AA patient samples within key genes, including TP73 (tumor suppression), XYLB (metabolism), ALDH4A1 (oxidative stress), PTPRB (cellular signaling), and HLA-DRB5 (immune response). These genetic variations likely contribute to disease progression and therapy response disparities.

This study highlights the importance of considering genetic and epigenetic variations in developing tailored therapeutic approaches to improve treatment efficacy and reduce mortality rates across diverse populations.

## Highlights

• Unique molecular signatures identified in African American prostate cancer patients, featuring distinct immune response pathways and genetic variants.
• Epigenetic regulation through histone modifications plays a crucial role in racial disparities.
• Over 2500 differentially expressed genes discovered between African American and Caucasian samples.

## Graphical abstract

Graphic Abstract:.

**Figure:**
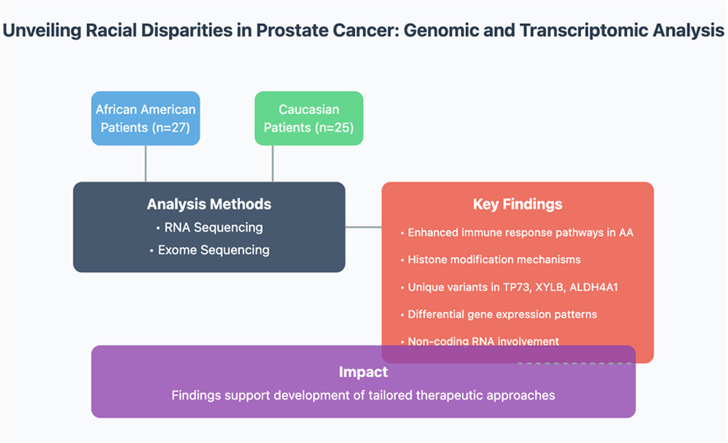

## 1 Introduction

Prostate cancer remains a significant public health concern, exhibiting notable disparities in incidence, progression, and outcomes among diverse racial and ethnic groups. Of particular significance is the observation that individuals of African descent, constituting the black population, manifest a higher tendency for prostate cancer incidence and often face more aggressive disease trajectories compared to individuals of American descent, represented by the white population. The occurrence of prostate cancer is 64% greater in African-American men than in Caucasian men, with a mortality rate for prostate cancer that is 2.3 times higher among African-American individuals (Siegel et al., 2022).

These disparities have prompted a spurred investigation aimed at understanding the underlying biological, environmental, and socioeconomic factors contributing to this phenomenon. Studies have identified both genetic and non-genetic factors that contribute to the higher incidence and aggressiveness of prostate cancer in African American men.

Genetic predisposition is a pivotal factor, with Genome-Wide Association Studies (GWAS) identifying Single Nucleotide Polymorphisms (SNPs) linked to increased prostate cancer risk, some of which show higher prevalence in Black cohorts. For instance, SNP rs577623 near the MSMB gene is significantly associated with increased prostate cancer risk in Black men (Singh et al., 2017). Variations in the androgen receptor (AR) gene sequence are also implicated in the more aggressive disease trajectory observed in Black men, with specific AR polymorphisms correlating with poorer clinical outcomes (Haiman et al., 2011).

Environmental and lifestyle factors, such as dietary habits, contribute to these disparities. Diets high in saturated fats and low in fruits and vegetables are more prevalent in Black communities, potentially increasing prostate cancer rates (Hayes et al., 1999). Socioeconomic determinants further compound these disparities, as Black men often face limited access to healthcare and preventative screenings, resulting in delayed diagnoses and suboptimal treatment options (Underwood et al., 2004).

Environmental and socio-economic factors exacerbate these health disparities. African Americans frequently face greater exposure to environmental pollutants and have historically had limited access to educational and healthcare resources, factors that contribute to the higher cancer rates observed in this group (Zavala et al., 2021). Additionally, differences in immune response and inflammation, as suggested by Wallace et al. (2008) (Wallace et al., 2008), indicate that biological responses to prostate cancer may also vary by race, potentially influencing the aggressiveness of the disease.

Collectively, these findings underscore the multifactorial nature of prostate cancer disparities. A comprehensive understanding of these factors is essential for developing targeted interventions and personalized treatment strategies that can help reduce these disparities and improve outcomes for African American men affected by prostate cancer.

### Bridging the gap: A comprehensive multi-omics approach

While existing research provides valuable insights, a critical gap remains in integrating advanced genomic and transcriptomic profiling to elucidate the precise biological pathways and gene expressions driving the observed disparities. This study addresses this gap by leveraging RNA-seq and exome sequencing.

Our study leverages RNA-seq and exome sequencing to identify unique genetic mutations and expression profiles in prostate cancer samples from African American and Caucasian men, to provide a more comprehensive understanding of the disease's molecular basis. This integrative approach allows us to explore how genetic and epigenetic factors, combined with non-genetic influences such as diet and environment, culminate in the observed disparities.

## 2 Materials and methods

### 2.1 Patient demographics

Our study included a total of 52 prostate cancer samples from both African American and Caucasian men, with data derived from RNA-seq and exome sequencing analyses from OSUCCC The ORIEN Avatar Research Program. The Oncology Research Information Exchange Network (ORIEN) is a unique alliance to integrate “big data” and data sharing for cancer research and care. ORIEN was founded by The Ohio State University Comprehensive Cancer Center and Moffitt Cancer Center in 2014 and now includes 18 members. The ORIEN Avatar Research Program generates prospective molecular (NGS) data on patients which is linked to longitudinal clinical data. These samples were meticulously selected to ensure a balanced representation, allowing for a robust comparative analysis of genetic and transcriptomic variations between the two groups.

The patient cohort consists of 27 samples from African American men and 25 samples from Caucasian men, ensuring a balanced representation for robust comparative analysis. Specifically, the RNA-seq data includes 14 samples from African American men and 12 from Caucasian men, while the exome sequencing data comprises 13 samples each from African American and Caucasian men.

**Fig. 1:**
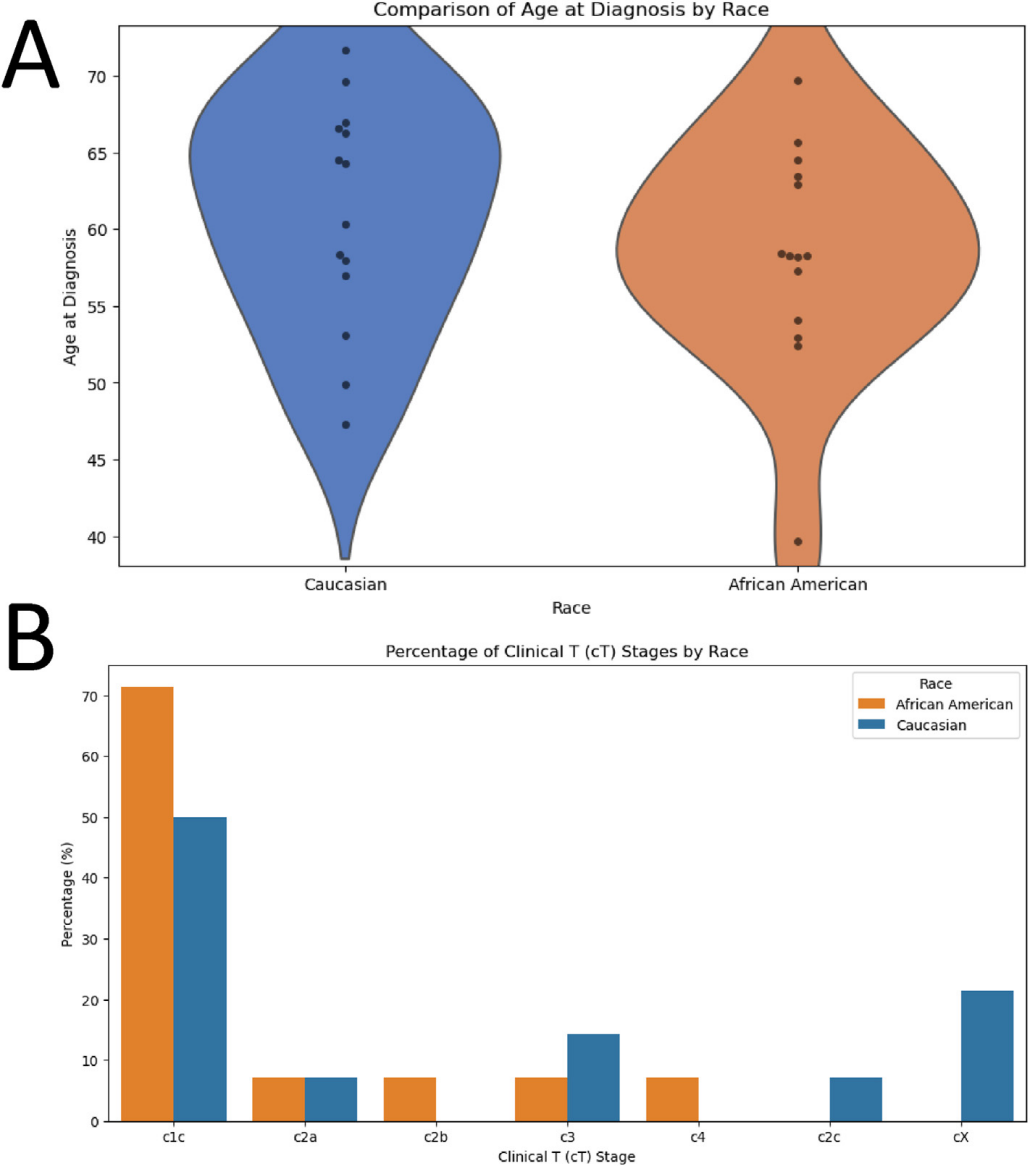
**Comparison of** a**ge at** d**iagnosis and Clinical T (cT)** s**tages by** r**ace in** p**rostate** c**ancer** s**amples.** (A) Violin plots with overlaid swarm plots (left) and box plots (right) showing the age distribution at diagnosis for African American (*n* = 14) and Caucasian (*n* = 14) patients. The groups show similar distributions (mean ± SD: African American 58.3 ± 7.3 years; Caucasian 61.0 ± 7.4 years) with no statistically significant difference (*t*-test, *p* = 0.34). Both groups showed normal distribution (Shapiro-Wilk test, *p* > 0.05) and equal variances (Levene's test, *p* = 0.52). The effect size was small to medium (Cohen's *d* = 0.37). (B) Bar plot presents the percentage distribution of Clinical T (cT) stages among Caucasian and African American prostate cancer patients. Clinical T stages indicate the extent and size of the primary tumor, ranging from c1c (small localized tumors) to c4 (large, locally advanced tumors) and cX (tumors that cannot be assessed).

The analysis of patient demographics revealed no distinct differences between Black and White prostate cancer patients regarding their age at diagnosis and the clinical stage of their tumors (Fig. 1).

The violin plot (Fig. 1(A)) illustrating the age at diagnosis shows that the median age is similar for both groups, with African American patients displaying a slightly narrower age range.

Further examination of the Clinical T (cT) stage distribution is shown depicted in (Fig. 1(B)), The percentage of African American patients diagnosed at the c1c stage have higher percentage compared to Caucasian patients.

Conversely, the cX stage, where the primary tumor cannot be assessed, is more among Caucasian patients, accounting for around 25% of cases. Both racial groups exhibit similar percentages for other stages, such as c2a and c2b, with only minor variations.

### 2.2 RNA sequencing data analysis

#### 2.2.1 Differential gene expression analysis (DEGs)

RNA sequencing (RNA-seq) data from prostate cancer samples were analyzed to identify differentially expressed genes (DEGs) between African American and Caucasian patient samples.

Quality Control and Pre-processing:

FastQC (v0.11.9) (Andrews, 2010) was used to assess raw sequencing data quality. Cutadapt (Martin, 2011) was then employed to trim adapter sequences and eliminate low-quality reads.

#### 2.2.2 Mapping to reference genome

Cleaned reads were aligned to the human reference genome using Hisat2 (Kim et al., 2015), ensuring accurate RNA-seq read mapping for reliable gene expression quantification.

#### 2.2.3 Quantification of gene expression

HTSeq-count (Anders et al., 2015) was utilized to count the reads aligning to each gene, generating count tables for DEG analysis.

#### 2.2.4 Normalization and differential expression analysis

Normalization of count data was performed to account for library size and sequencing depth variations. DESeq2 (Love et al., 2014) was then used was then used to identify genes differentially expressed between the two ethnic groups.

**Principal Component Analysis (PCA)** (R Core Team R, 2013), volcano plots(Blighe et al., 2019), and heatmaps (Kolde, 2019) were generated using R (v4.1.0) to visualize gene expression patterns and identify DEGs.

***Filtering of Race-Specific DEGs*:** Similarly, we identified DEGs in African American prostate cancer samples compared to Caucasian samples, and vice versa. To exclude race-specific DEGs, we utilized data from GSE6956, which includes prostate tumor samples from both African American and Caucasian patients, as well as surrounding normal prostate tissue from these racial groups. The surrounding normal prostate tissue from African American and Caucasian individuals served as controls to filter out race-specific DEGs, allowing us to focus on genes differentially expressed in cancer that are not influenced by racial differences.

### 2.3 Exome sequencing data analysis

#### 2.3.1 Variant identification

Quality Control and Pre-processing:

FastQC (v0.11.9) (Andrews, 2010) and Cutadapt (Martin, 2011) were employed for quality assessment and read trimming, respectively.

#### 2.3.2 Read mapping

Cleaned reads were mapped to the human reference genome (hg38) using BWA-MEM. During mapping, the 'ID' (identifier) and 'SM' (sample name) tags were included to facilitate sample identification and management in downstream analyses, followed by post-mapping processing steps to refine alignments.

#### 2.3.3 Mapped reads postprocessing

After initial mapping, reads were rigorously filtered using SAMtools (Barnett et al., 2011) to ensure only those with a mate mapped and a minimum mapping quality score of at least 1 were retained. Duplicate reads were subsequently removed with Picard MarkDuplicates to avoid biases in variant calling. The FreeBayes suite's BamLeftAlign (Garrison & Marth, 2012) was utilized to realign indels, enhancing the accuracy of variant detection. Read mapping qualities were recalibrated using SAMtools calmd (Li, 2013) to update Base Alignment Quality (BAQ), which is critical for precise SNP and indel calling. A final filtering step was applied to exclude any reads with recalibrated mapping quality scores exceeding 254, ensuring only the highest quality data was used for further analysis (Barnett et al., 2011).

#### 2.3.4 Variant calling

Variants were called using GATK's HaplotypeCaller (McKenna et al., 2010) to identify SNPs and insertions/deletions (indels) that could potentially influence prostate cancer characteristics and outcomes (McKenna et al., 2010).

#### 2.3.5 Variant comparative analysis

A Python-based comparative analysis detected unique variants in African American samples compared to Caucasian samples, aiming to identify genetic differences contributing to prostate cancer disparities.

#### 2.3.6 Variant annotation and gene impact analysis

Variants were annotated using SnpEff (Marchitti et al., 2008) to understand their biological impact. Special attention was given to high-impact mutations, including loss of function mutations such as stop gained, stop lost, start lost, and missense mutations. These mutations were prioritized as they are more likely to alter protein function significantly and contribute to disease pathology.

### 2.4 Integration with RNA-seq data

In the integration phase, genes identified with high-impact and loss of function mutations during variant annotation were cross-referenced with RNA-seq data to assess and compare expression levels between African American and Caucasian samples.

#### 2.4.1 Filtering race-specific variants

***Annotation of****u****nique****v****ariants:*** Unique variants in African American prostate cancer samples compared to Caucasian samples, and vice versa, were annotated using the Variant Effect Predictor (VEP). This tool provided comprehensive information on the effects and annotations of the variants, including specific allele frequencies (AF) for different racial backgrounds.

***Filtering****v****ariants by****a****llele****f****requency in African American****p****atients:*** Variants specific to African American patients were filtered based on their allele frequencies. Variants with allele frequencies found in healthy African American individuals (AF < 0.01) were excluded, allowing us to focus on variants potentially unique to the cancer cohort.

***Filtering****v****ariants by****a****llele****f****requency in Caucasian white****p****atients:*** Similarly, variants in Caucasian patients were filtered by allele frequency. Variants with allele frequencies present in healthy Caucasian individuals (AF < 0.01) were excluded, identifying those unique to the cancer cohort.

## 3 Results

### 3.1 RNA-seq analysis

RNA-seq analysis was conducted on a total of 26 prostate cancer samples, comprising 14 samples from Caucasian white men and 12 samples from African American men. The methodology, as outlined in the methods section, ensured a comprehensive examination of the transcriptomic profiles associated with prostate cancer in these distinct demographic groups.

The raw sequencing data were processed and filtered to remove low-quality reads, and subsequent alignment to the reference genome was performed using Hisat2. Following alignment, the expression levels of genes were quantified, and differential gene expression analysis was carried out using Deseq2. The criteria for significance were set at a fold change of at least 1.5 and an adjusted p-value below 0.01.

**Fig. 2:**
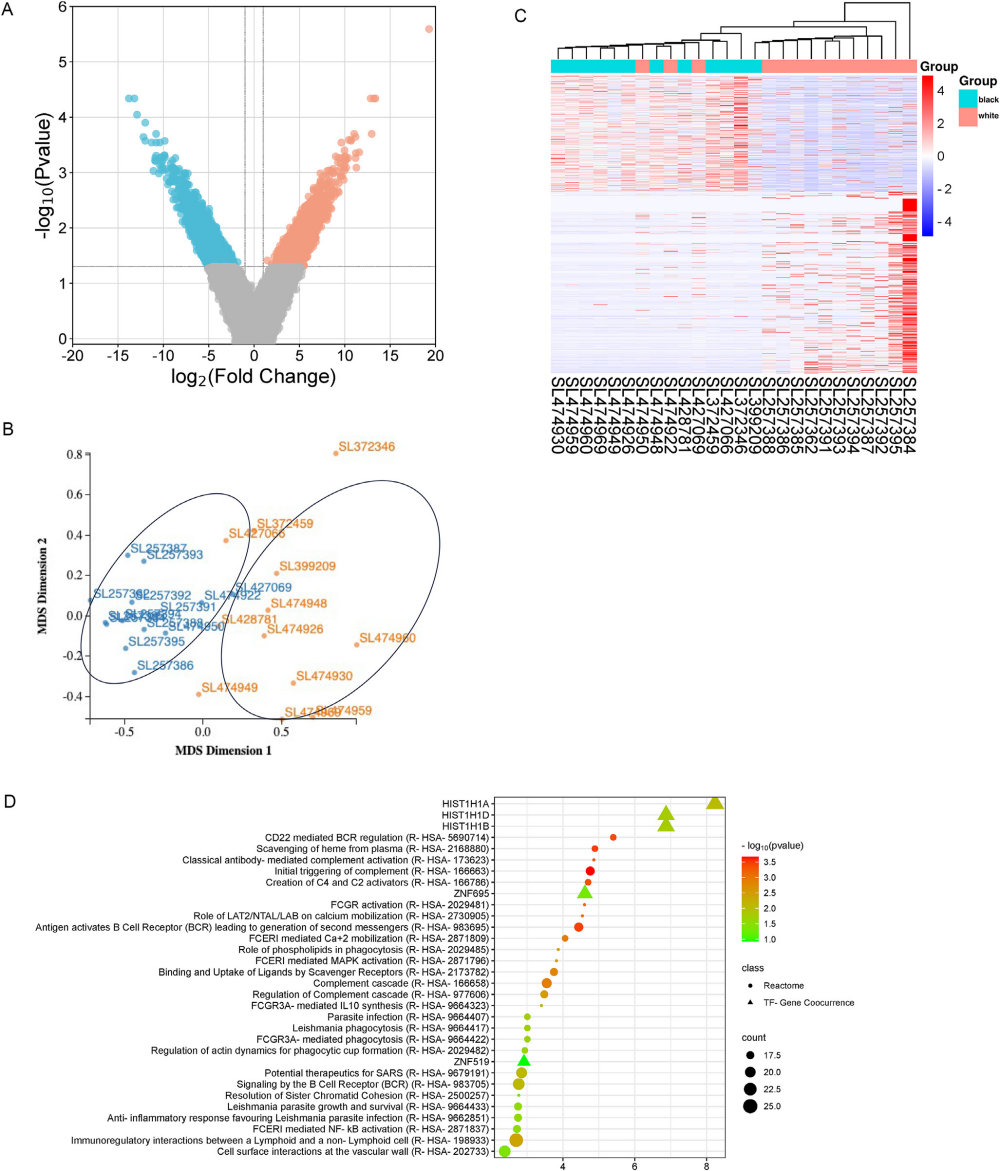
**Integrative** a**nalysis of** d**ifferentially** e**xpressed** g**enes (DEGs) in** p**rostate** c**ancer.** (A) Volcano plot of DEGs: The upper left panel displays a volcano plot representing the distribution of genes based on fold change and statistical significance in prostate cancer samples from African American men compared to Caucasian white men. (B) Multidimensional scaling (MDS) plot: The lower panel features an MDS plot illustrating the multidimensional relationships between prostate cancer samples from the two ethnic groups. Each point represents an individual sample, with distances indicating the overall similarity or dissimilarity in gene expression patterns. Samples from African American men are shown in orange and samples from Caucasian white men are shown in blue. Distinct clustering reveals pronounced differences in transcriptomic profiles between African American and Caucasian white men. (C) Hierarchical clustering heatmap of DEGs: The right panel showcases a hierarchical clustering heatmap depicting the expression profiles of DEGs in prostate cancer samples from African American men compared to Caucasian white men. (D) Enrichment analysis plot illustrating Reactome pathways of upregulated differentially expressed genes (UP DEGs) in prostate cancer samples isolated from African American men, compared to white Caucasian counterparts.

To visualize the global expression patterns and identify DEGs, volcano plots and MDS plots were generated (Fig. 2). The MDS plot provides a multidimensional representation of the samples, enabling the identification of clustering patterns and potential outliers. Notably, the MDS plot (Fig. 2(B)), revealing two distinct groups, indicates obvious differences in gene expression levels between samples isolated from African American and Caucasian white men. This observation underscores the genetic diversity and potential biological distinctions in prostate cancer between the two ethnic groups. The volcano plot (Fig. 2(A)) displays the relationship between fold change and statistical significance for each gene, with upregulated genes on one side, downregulated genes on the other, and significant DEGs highlighted.

The analysis revealed a total of 1003 upregulated genes and 1589 downregulated genes in samples isolated from prostate cancer of African American men compared to white Caucasian men (Fig. 2(A)–(C)). To further elucidate the gene expression patterns, we generated a hierarchical clustering heatmap (Fig. 2(C)) depicting the upregulated and downregulated DEGs in samples isolated from African American men compared to Caucasian white men. Notably, the hierarchical clustering demonstrated clear distinctions in the transcriptional landscape between the two ethnic groups.

The enrichment analysis of UP DEGs in prostate cancer samples from African American men, compared to white Caucasian counterparts, revealed significant upregulation in Reactome pathways associated with diverse cellular processes. Notably, enriched pathways include those related to phagocytosis, immune response, complement cascade, and cellular interactions (Fig. 2(D)), highlighting the potential involvement of these pathways in prostate cancer disparities. Additionally, we conducted an enrichment analysis using the TF-Gene Cooccurrence database to explore transcription factor (TF) interactions. Intriguingly, HIST1H1A, HIST1H1D, and HIST1H1B emerged prominently in the analysis, suggesting their involvement in transcriptional regulation. These genes are associated with histones, indicating a potential role of histone modifications and epigenetic regulation in the observed gene expression changes (Fig. 2(D)). In contrast, the majority of downregulated genes were found to be pseudogenes and long non-coding RNA, while among the upregulated genes, there were also pseudogenes and long non-coding RNA. The enrichment of pseudogenes and long non-coding RNA suggests potential involvement of non-coding elements in the observed gene expression changes, indicating a possible role of epigenetic factors in shaping the molecular landscape of prostate cancer disparities.

**Fig. 3:**
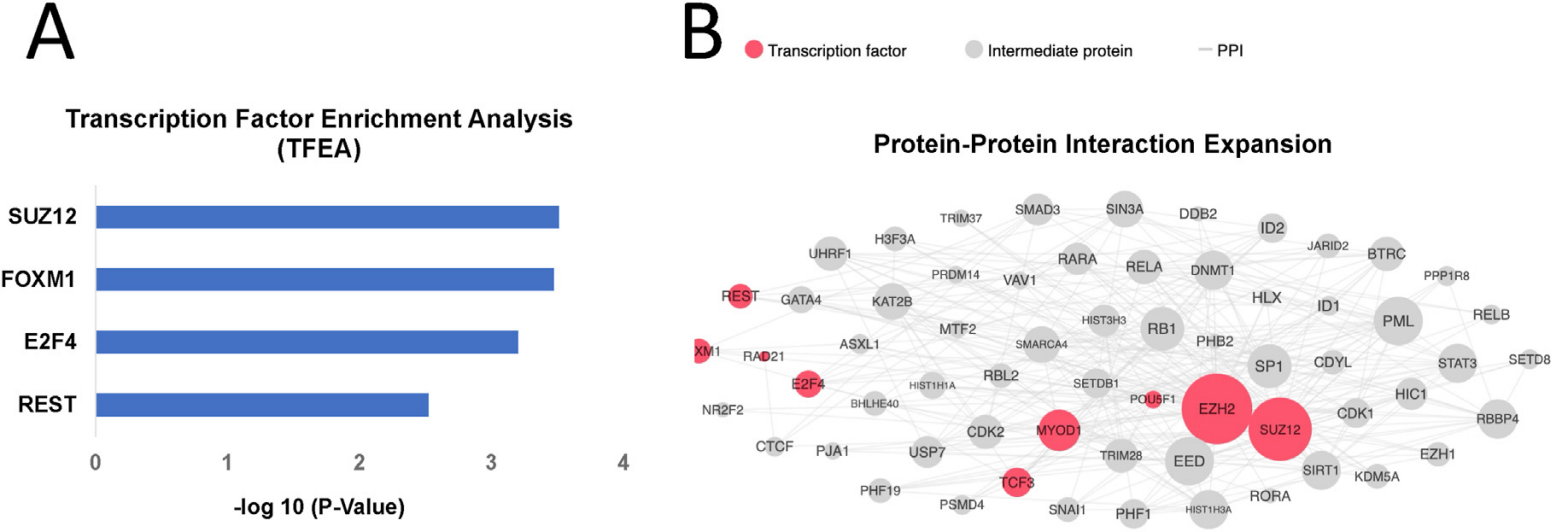
**X2K analysis of UP DEGs in AA prostate cancer samples.** (A) The bar chart represents the results of Transcription Factor Enrichment Analysis (TFEA) for UP DEGs in prostate cancer samples from African American patients compared to Caucasian white patients. The *x*-axis indicates the transcription factors, while the *y*-axis shows the -log_10_ (*P*-value) of the enrichment scores, highlighting the significance of each transcription factor in the context of racial disparities in prostate cancer. (B) The protein-protein interaction expansion plot illustrates the interaction network of the UP DEGs in prostate cancer samples from African American patients compared to Caucasian white patients.

Furthermore, X2K (eXpression2Kinases) analysis (Clarke et al., 2018) of UP DEGs in AA prostate cancer samples showed enrichment of EZH2 and SUZ12 transcription factors which are elements of PRC2 complex (Margueron & Reinberg, 2011) (Fig. 3). These findings align with a recently published research by Ramakrishnan et al. (Ramakrishnan et al., 2024), which showed that DNA hypermethylated regions are enriched for PRC2/H3K27me3 pathways and EZH2/SUZ12 cofactors. This parallel underscore the potential significance of epigenetic regulation, particularly involving SUZ12 and EZH2, in contributing to prostate cancer disparities among different racial groups.

### 3.2 Filtering race-specific DEGs

**Fig. 4:**
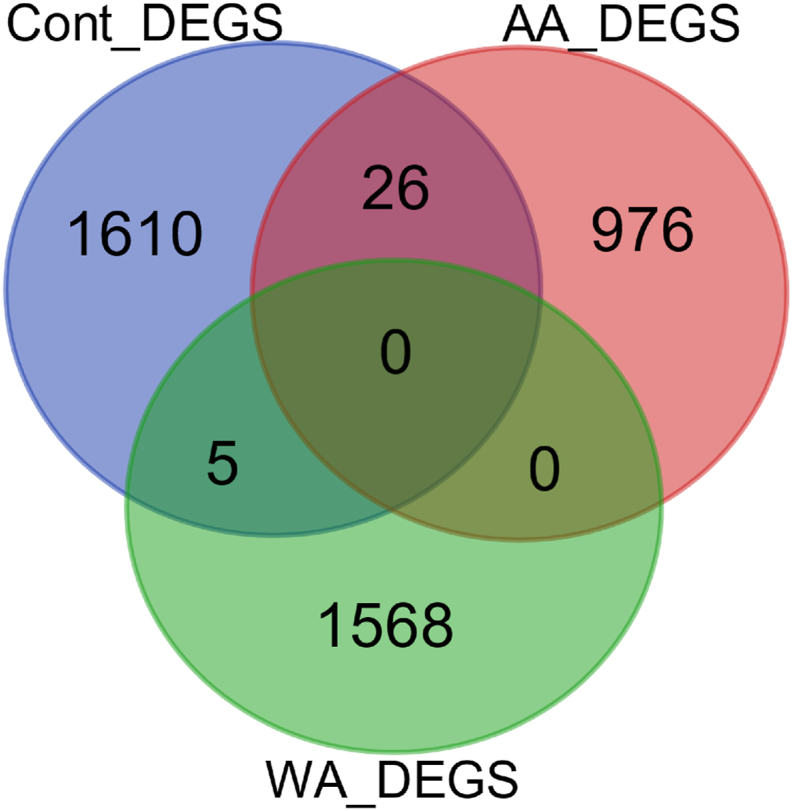
**Comparison of DEGs among african American and Caucasian prostate cancer samples and healthy prostate tissue.This Venn diagram illustrates the overlap of DEGs identified in African American (AF) prostate cancer samples, Caucasian white (WA) prostate cancer samples, and healthy prostate tissue from both racial groups. The diagram highlights the distinct nature of the identified DEGs, with minimal overlap observed, indicating that the majority of DEGs between African American and Caucasian prostate cancer samples are not influenced by variations in healthy prostate tissue. Each circle represents the unique and shared DEGs across the groups, demonstrating the specific gene expression profiles associated with prostate cancer in each racial cohort.**

In this study, we compared data from African American and Caucasian prostate cancer samples. To exclude race-specific DEGs, we utilized data from GSE6956, which includes prostate tumor samples from both African American and Caucasian patients, as well as surrounding normal prostate tissue from these racial groups. The DEGs between the surrounding normal prostate tissue from African American and Caucasian individuals served as controls to filter out race-specific DEGs, allowing us to focus on genes differentially expressed in cancer that are not influenced by racial differences (Fig. 4).

The Venn diagram provides a visual representation of the DEGs identified in African American (AF) and Caucasian white (WA) prostate cancer samples, alongside the DEGs found in healthy prostate tissue from both racial groups. The diagram reveals that there is minimal overlap among the three sets of DEGs, indicating that most of the identified DEGs in the cancer samples are distinct from those in healthy tissues. This lack of significant overlap suggests that the biological mechanisms driving cancer progression may differ between these populations, underscoring the importance of considering racial factors in cancer genomics and treatment strategies.

### 3.3 Exome sequencing analysis

#### 3.3.1 Unique mutations in African American patients

**Fig. 5:**
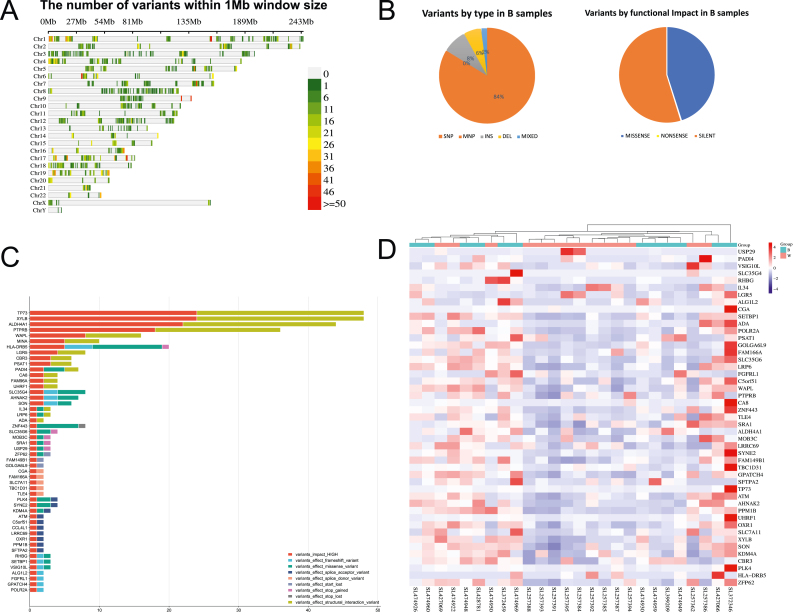
**Genomic and** e**xpression** l**andscape of African American** p**rostate** c**ancer.** (A) Genomic density plot revealing chromosomal distribution of somatic variants across 1 Mb bins, highlighting mutational hotspots and regions of genomic instability. (B) Pie charts quantifying the spectrum of mutation types, with functional classification (left) and predicted genomic impact (right) in African American samples. (C) Bar plot summarizing mutation frequencies in significantly altered genes, encompassing high-impact variants, structural variants, and loss-of-function mutations (frameshift, stop-gained/lost, start gain, missense, and splice site variants). (D) Expression heatmap revealing transcriptional patterns of mutation-affected genes in African American prostate cancer samples relative to Caucasian samples, demonstrating population-specific expression signatures.

Exome sequencing analysis revealed 5419 mutations unique to prostate cancer samples from African American patients compared to Caucasian white patients. These mutations represent distinctive genetic alterations present in prostate cancer among individuals of African descent. In-depth analysis of these mutations was conducted to elucidate their functional consequences and potential implications for prostate cancer biology. Fig. 5(A) illustrates the mutation density across the genome, with each chromosome divided into bins to assess the distribution of variants. The majority of mutations were found to be silent mutations (54.78%), followed by missense mutations (45.07%), with a minimal percentage of nonsense mutations (0.16%) (Fig. 5(B)). Further analysis of mutation types was performed to assess their relative impact. Fig. 5(B) illustrates the proportion of each mutation type, highlighting the prevalence of single nucleotide polymorphisms (SNPs) (83.74%), followed by insertions (INS) (8.24%), deletions (DEL) (5.93%), multiple nucleotide polymorphisms (MNP) (0.05%), and mixed variants (MIXED) (2.03%).

#### 3.3.2 Identification of affected genes in african American patients

The affected genes associated with these mutations were identified using SNPeff, revealing a total of 2675 genes affected by different types of mutations (supplemental materials). These mutations encompassed a range of functional impacts, including missense variants, frameshift variants, stop-gained variants, and others (supplemental materials).

In our analysis, we specifically focused on variants with significant effects on genes, including high-impact variants, structural interaction variants, and loss-of-function mutations such as frameshift variants, stop-gained, stop lost, start gain, missense, splice acceptor, and splice donor variants. This targeted approach allowed us to identify alterations that could profoundly influence gene function and potentially contribute to disease pathogenesis. Fig. 5(C) illustrates the distribution and frequency of mutations in genes affected by these types of mutations, providing valuable insights into their prevalence and potential implications for disease mechanisms.

We utilized RNA sequencing data from African American cancer samples and Caucasian white samples to examine the gene expression of those genes affected by mutations identified in Fig. 5(C) and Fig. 5(D), showing a heatmap of the expression levels of these genes. Importantly, our analysis revealed that the majority of these genes were downregulated in African American samples, suggesting potential disparities in gene expression patterns between racial groups.

The distribution and frequency of mutations across the most affected genes (top 50 genes) in prostate cancer samples from African American patients are shown in Fig. 5(D), highlighting the diverse array of mutations and affected genes associated with the disease (see Table 1).

#### 3.3.3 Unique mutations in Caucasian white patients

**Fig. 6:**
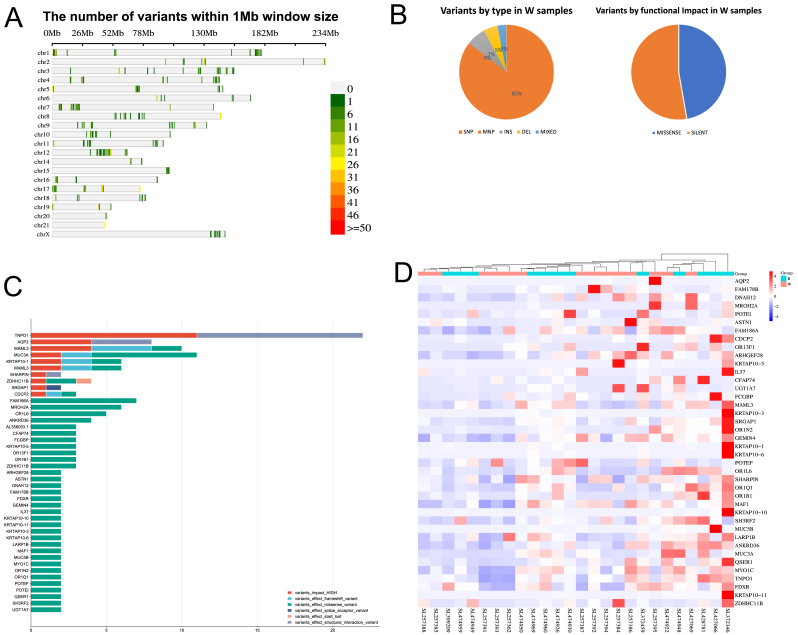
**Analysis of** m**utation** t**ypes and** g**ene** e**xpression in Caucasian white** p**rostate** c**ancer** s**amples.** (A) This panel illustrates the mutation density across the genome, with each chromosome divided into bins to assess the distribution of variants. (B) Pie charts show the distribution of different mutation types based on functional classification and based on their impact. (C) Bar plot displays the distribution and frequency of mutations in genes affected by high-impact variants, structural interaction variants, and loss-of-function mutations such as frameshift variants, stop-gained, stop lost, start gain, missense, splice acceptor, and splice donor variants. Each bar represents one gene. (D) Expression heatmap presents a heatmap showing the gene expression levels of the identified genes in Caucasian white cancer samples compared to african American samples. The heatmap highlights the expression patterns of genes affected by mutations identified in the analysis.

On the other hand, Caucasian white samples exhibited 1146 unique mutations (Table 2) (Fig. 6(A)), revealing distinct genetic alterations in prostate cancer among individuals of European descent. The majority of mutations were silent (52.75%) and missense (47.25%), with minimal percentages of other mutation types. Single nucleotide polymorphisms (SNPs) were the most prevalent mutation type (85.36%), followed by insertions (7%) and deletions (5%) (Fig. 6(B)). These findings shed light on the unique mutation profiles in prostate cancer among Caucasian white patients.

**Table 1:** Distribution of subjects by type and ethnicity.

Type	Ethnicity	Count
RNAST	Caucasian patient	12
RNAST	African American	14
Exom	Caucasian patient	13
Exom	African American	13

**Table 2:** Functional annotation of high-impact mutations.

Gene	Function	Variant Type	Biological Impact	Pathway Involvement
TP73	Tumor Suppression	Missense	Disrupts Tumor Suppression	Cell Cycle Regulation
XYLB	Metabolism	Frameshift	Impairs Metabolic Pathways	Carbohydrate Metabolism
ALDH4A1	Oxidative Stress	Stop-Gained	Compromises Oxidative Stress Response	Detoxification Processes
PTPRB	Cellular Signaling	Splice Acceptor	Alters Cellular Signaling	Receptor Tyrosine Kinase Signaling
HLA-DRB5	Immune Response	Stop-Lost	Impacts Immune Response	Antigen Presentation

#### 3.3.4 Identification of affected genes in Caucasian white patients

Similar to Black samples, Caucasian white samples underwent a focused analysis to identify variants significantly affecting genes. Fig. 5(C) displays the distribution and frequency of mutations in genes affected by these variants, providing crucial insights into their prevalence and potential disease implications.

Utilizing RNA sequencing data from Caucasian white cancer samples, we examined the gene expression of those affected genes identified in Fig. 6(C). Fig. 6(D) presents a heatmap of their expression levels, revealing important insights into gene expression patterns in Caucasian white samples. This underscores the impact of mutations on gene expression profiles in the Caucasian white patient cohort.

### 3.4 Genes with high-impact mutations in african American prostate cancer samples

We first identified unique variants in each group and then determined the genes impacted by these mutations. Among these, TP73, XYLB, ALDH4A1, PTPRB, and HLA-DRB5 exhibited high-impact mutations in African American prostate cancer samples, compared to Caucasian white samples Table 2.

TP73, a member of the tumor suppressor p53 family, plays critical roles in cell cycle regulation, apoptosis, and genomic stability (Stiewe & Pützer, 2000). Alterations in this gene could disrupt these pathways, potentially leading to more aggressive cancer phenotypes and influencing patient prognosis in prostate cancer. Similarly, the gene HLA-DRB5, which plays a significant role in the immune response, shows unique variants that could be implicated in the immune system's ability to recognize and respond to tumor cells. This gene's variants might affect the presentation of tumor antigens and modulate the immune surveillance of prostate cancer cells, potentially leading to differences in cancer progression and outcomes between populations (Chowell et al., 2018).

ALDH4A1, part of the aldehyde dehydrogenase family, is crucial for oxidative stress response. Variants in ALDH4A1 may modify the cellular response to oxidative stress in tumor environments, affecting growth rates and resistance to therapies (Marchitti et al., 2008). PTPRB, encoding a protein tyrosine phosphatase, regulates critical signaling pathways that influence growth factor signaling, cell cycle progression, and migration—key processes in cancer metastasis and progression (Huang et al., 2018).

Furthermore, the gene XYLB is involved in carbohydrate metabolism, might affect cancer metabolism, supporting the rapid growth characteristic of cancer cells, although its direct links to prostate cancer are less established (Pavlova & Thompson, 2016).

### 3.5 Filtering race-specific variants

In this study, we compared data from African American and Caucasian prostate cancer samples. To exclude variants due to race, we performed additional filtering of the identified mutations in each group to focus solely on mutations related to cancer samples, as detailed in the methods section.

**Fig. 7:**
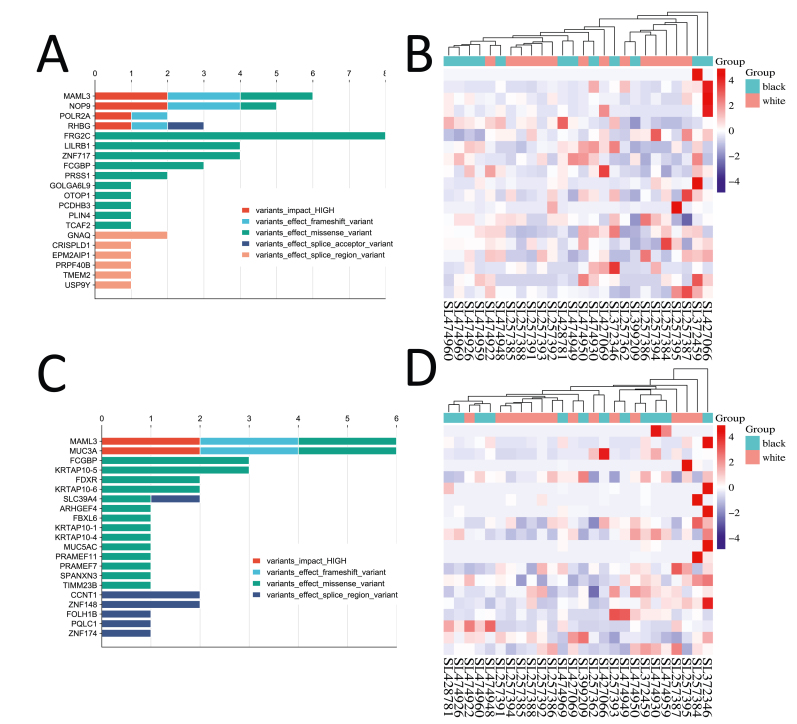
**Distribution andexpression ofmutationspost-race-specificfiltering**. Panels (A) and (C) illustrate the distribution and frequency of mutations in genes impacted by high-impact variants, structural interaction variants, and loss-of-function mutations, specifically after filtering out race-specific mutations in each group. Each bar in these figures represents a unique gene, with panel (A) focusing on mutations identified in African American samples, while panel (C) highlights those found in Caucasian samples. Panels (B) and (D) provide heatmaps that depict the gene expression levels of the affected genes in the respective cancer samples. Panel (B) showcases the expression patterns of genes associated with the identified mutations within the African American cohort, whereas panel (D) details the gene expression levels in the Caucasian cohort.

After filtering out race-specific variants, we identified 266 unique mutations in African American prostate cancer samples. These mutations affected 20 genes (Fig. 7(A)–(C)) and included high-impact variants, structural interaction variants, and loss-of-function mutations, such as frameshift variants, stop-gained, stop-lost, start gain, missense, splice acceptor, and splice donor variants. In contrast, we found 415 unique mutations in Caucasian samples following the same filtering process, which impacted 21 genes (Fig. 7(B)–(D)).

## 4 Discussion

Our comprehensive analysis integrating RNA-seq and exome sequencing data provides new insights into the molecular underpinnings of prostate cancer disparities between African American and Caucasian men. By identifying unique genetic variants and differential gene expression profiles, our findings underscore the complex interplay of genetic, epigenetic, and environmental factors in driving these disparities.

Our study identified unique variants in both African American and Caucasian prostate cancer samples. Key genes with high-impact mutations predominantly found in African American samples include TP73, XYLB, ALDH4A1, PTPRB, and HLA-DRB5. These genes are involved in critical biological processes such as tumor suppression, metabolism, oxidative stress, cellular signaling, and immune response. This aligns with prior research by Singh et al. (2017) (Singh et al., 2017), which highlighted significant SNPs associated with prostate cancer risk specifically in African American men.

The pathway enrichment analysis revealed upregulated pathways linked to immune response and cellular interactions, notably involving histone-associated transcription factors such as HIST1H1A, HIST1H1D, and HIST1H1B. These findings suggest that epigenetic modifications could play a pivotal role in the observed gene expression changes, influencing tumor behavior and patient prognosis. This complements the study by Wallace et al. (2008) (Wallace et al., 2008), which emphasized differences in inflammatory responses and immune system activities between racial groups.

The prevalence of pseudogenes and long non-coding RNA among the regulated genes particularly in Caucasian white prostate samples further highlights the potential involvement of non-coding elements in prostate cancer disparities. These elements could affect gene regulation and epigenetic states, contributing to the distinct molecular profiles seen in African American patients. This finding aligns with the studies by lee et al. (2024) (Lee et al., 2024) and yuan et al. (2020) (Yuan et al., 2020) which also identified lncRNAs as a significant component of the transcriptomic differences between African Ancestry (AA) and European Ancestry (EA) individuals.

A recent study revealed the involvement of keratin family genes in African American prostate cancer samples (Mori et al., 2022). Similarly, in my study, genes such as KRT13, KRT18P18, KRT18P34, KRT18P63, KRT7-AS, KRT87P, KRT8P15, KRT8P31, KRT8P39, KRT8P46, KRT8P9, KRTAP10-11, and KRTAP10-9 were identified as significantly upregulated. Conversely, in the other study (Mori et al., 2022), KRT8, KRT15, KRT19, KRT34, and KRT80 were found to be enriched in African American prostate cancer samples. These findings underscore the intricate diversity of keratin expression patterns observed in prostate cancer among different racial groups.

Our X2K analysis unveiled heightened expression of EZH2 and SUZ12, core components of the PRC2 complex, in AA prostate cancer. This underscores the potential involvement of epigenetic regulation, particularly implicating these factors, in contributing to racial disparities. This observation is consistent with recent findings by Ramakrishnan et al. (Ramakrishnan et al., 2024), which highlighted the enrichment of EZH2 and SUZ12 in DNA hypermethylated regions.

In addition to genetic and epigenetic factors, socioeconomic and environmental influences remain critical. As Hayes et al. (1999) (Hayes et al., 1999) have noted, factors such as dietary habits, healthcare access, and environmental exposures significantly impact cancer risk and outcomes. Our findings should be viewed within the context of these broader determinants, which continue to shape health disparities.

Understanding the molecular basis of prostate cancer disparities is essential for developing targeted interventions. Our study highlights the importance of personalized therapeutic strategies that account for genetic and epigenetic diversity. Future research should focus on functional studies to validate the role of the identified genes and pathways and explore how these insights can be translated into clinical practice.

While our study provides significant insights, it is limited by the sample size and the focus on specific populations. Larger, more diverse cohorts and longitudinal studies are necessary to confirm these findings and understand their broader applicability. Most importantly, it would be essential to validate biological associations between these genomic and transcriptomic landscapes among black and white patients and disparities of prostate cancer using patient-derived models including cell models and xenograft models. Traditionally, cancer research and drug development utilize cancer cell lines(Daniel et al., 2009) (McDermott et al., 2007) (Barretina et al., 2012) (Johnson et al., 2001) (Izumchenko et al., 2016), and their derived cancer models. The three cell lines used most frequently are PC-3, DU 145 and LNCaP, these were all derived from metastases, and therefore are not representative of primary PCa (Peehl, 2005). A complication to use these lines is that continuous passaging of such cell lines *in vitro* leads to genetic drift due to further acquired genetic alterations(Wilding & Bodmer, 2014). In recognition of the many limitations of these cancer models, patient-derived models of cancer (PDMCs) were developed recently, including patient-derived xenografts (PDXs), conditionally reprogrammed cell cultures (CRCs), organoids, spheroids, induced pluripotent stem cells (iPSC) and others (Jin et al., 2010; Tentler et al., 2012) (Pan et al., 2015; Williams et al., 2013) (Boj et al., 2015; Drost et al., 2015, 2016; Gao et al., 2015; Karthaus et al., 2015; Li et al., 2014; Nadauld et al., 2014; Sachs & Clevers, 2014; van de Wetering et al., 2015). CR and organoid cultures represent the next generation of *in vitro* patient-derived cell models. These *in vitro* systems have been described and highlighted in the NCI precision medicine initiative and human cancer model initiative (HCMI). The CR technique is relatively simple and has been reproduced in more than 60 laboratories(Beglyarova et al., 2016; Boehm & Golub, 2015; Brown et al., 2015; Butler et al., 2016; Chu et al., 2015; Crystal et al., 2014; Friedman et al., 2015; Garraway & Lander, 2013; Hata et al., 2016; Prater et al., 2014; Saeed et al., 2016; Veit et al., 2016; Walters et al., 2015). To our knowledge, CR is the only technology that allows expansion of cell cultures from normal prostate, primary PCa, metastatic and/or castration resistant PCa, NEPC, and circulating tumor cells (CTC) from metastatic PCa patients (Armstrong et al., 2019; Liu et al., 2017; Saeed et al., 2016; Timofeeva et al., 2017). Our preliminary data indicated the higher sensitivity of normal AA prostate cells to Myc/T58A (a natural tumor-derived mutant c-Myc) compared that of normal EA prostate cells. Given the critical role of Myc in carcinogenesis and resistance to therapeutic intervention, we propose to develop relevant cell models from AA and EA populations to probe for differences in the sensitivity of normal prostate cells to androgens and their susceptibility to immortalization and transformation, very early steps of carcinogenesis. We are interrogating differences and mechanisms of the biological behaviors of primary and advanced PCa cells and PCa progression from AA and EA men *in vitro* and *in vivo*. These results will be critical for biological validation of the above genetic and epigenetic variations and developing tailored therapeutic approaches to improve treatment efficacy and reduce mortality rates across diverse populations.

## 5 Conclusion

In conclusion, our integrative analysis has unveiled a nuanced portrait of the genomic and transcriptomic landscapes in prostate cancer among black and white patients. These identified genetic mutations, altered gene expressions, and their convergence in specific pathways contribute to a deeper understanding of the molecular underpinnings of prostate cancer disparities. These findings may pave the way for more effective diagnostic approaches, targeted therapeutic interventions, and personalized treatment strategies, ultimately striving towards reducing racial disparities in prostate cancer outcomes.

## CRediT authorship contribution statement

**Abdalla Elbialy:** Writing – review & editing, Writing – original draft, Methodology, Investigation, Formal analysis, Data curation. **Akshay Sood:** Writing – review & editing, Supervision, Methodology, Investigation, Conceptualization. **Shang-Jui Wang:** Writing – review & editing, Methodology, Investigation, Conceptualization. **Peng Wang:** Writing – review & editing, Resources, Methodology, Investigation, Conceptualization. **Ahmed Fadiel:** Writing – review & editing, Methodology, Formal analysis. **Anil V. Parwani:** Writing – review & editing, Supervision, Resources, Methodology, Investigation, Conceptualization. **Steven Huang:** Writing – review & editing, Resources, Formal analysis. **Gennady Shvets:** Writing – review & editing, Validation. **Nagireddy Putluri:** Writing – review & editing, Investigation. **Jenny Li:** Writing – review & editing, Resources, Project administration, Investigation, Conceptualization. **Xuefeng Liu:** Writing – review & editing, Writing – original draft, Resources, Project administration, Investigation, Funding acquisition, Data curation, Conceptualization.

## Conflicts of interest

Several patents for CR technology have been awarded to 10.13039/100008064Georgetown University by the US Patent Office. The license for this technology has been given to a Maryland-based start-up company for commercialization. The inventor, X.L., and Georgetown University receive potential royalties and payments from the company. CR media and CR cells have been distributed by Propagenix (acquired by StemCell Technologies), Fisher Scientific, ATCC, etc. Other authors declare that they have no known competing financial interests or personal relationships that could have appeared to influence the work reported in this paper.
